# Exploring the n-back task: insights, applications, and future directions

**DOI:** 10.3389/fnhum.2025.1721330

**Published:** 2025-12-05

**Authors:** Shaojia Huang, Caini Chen, Yuanqin Mo, Yihan Zhao, Yuge Zhu, Kangli Dong, Tao Xu

**Affiliations:** 1 School of Intelligent Manufacturing and Aeronautics, Zhuhai College of Science and Technology, Zhuhai, China; 2 Department of Biomedical Engineering, Shantou University, Shantou, China

**Keywords:** n-back task, working memory, fluid intelligence, multi-cognitive process, mental fatigue

## Abstract

The n-back task has become a central paradigm for investigating the mechanisms of working memory (WM) and related executive functions. This review provides an integrative analysis of the n-back experiment, covering its cognitive operations, task variants, neuroimaging findings, and practical applications across multiple domains. We first delineate three core cognitive components—updating, maintenance, and attentional control—and summarize converging evidence that these functions rely on overlapping fronto-striatal and fronto-parietal networks. We then examine major task variants and review applications in: (1) cognitive training and transfer effects, particularly the proposed association between WM and fluid intelligence; (2) clinical contexts including attention deficit hyperactivity disorder (ADHD), depression, and neurological rehabilitation; (3) developmental and educational settings; and (4) emerging research on social cognition, stress, and emotional regulation. Critically, this review evaluates ongoing inconsistencies in how the n-back task is interpreted as a measure of WM and highlights methodological factors, such as task heterogeneity, multi-process interference, and mental fatigue, that complicate both behavioral and neural inferences. To address these issues, we outline methodological recommendations including adaptive task design, multimodal physiological monitoring, and standardized experimental protocols. We further discuss future directions involving virtual reality (VR), mobile platforms, and brain-computer interface (BCI) integration to improve ecological validity and translational relevance. By synthesizing behavioral and neural evidence, this review underscores the n-back task’s versatility while emphasizing the need for improved construct clarity and methodological rigor.

## 1 Introduction

WM is essential for the temporary storage and manipulation of information that supports reasoning, learning, and decision-making (Ribeiro et al., 2019). Among numerous paradigms used to study WM, the n-back task has become one of the most versatile and widely adopted approaches because it allows dynamic manipulation of cognitive load and continuous monitoring of neural activity. Originally introduced by Kirchner (1958), the paradigm requires participants to judge whether the current stimulus matches one presented n trials earlier, thereby engaging processes of updating, maintenance, and attentional control (Kirchner, 1958).

Many meta-analyses have examined the n-back paradigm from behavioral, neuroimaging, and training perspectives (Table 1), yet key methodological and interpretational inconsistencies remain unresolved. Existing reviews tend to focus on isolated aspects of the paradigm, whereas a unified synthesis integrating cognitive mechanisms, task variants, and multimodal neural evidence is still lacking. In addition, variability in task parameters, multi-process cognitive interference, and the often-overlooked impact of mental fatigue have contributed to inconsistent conclusions regarding what the n-back task measures and how its results should be interpreted. Clarifying these issues is essential for improving the validity, reliability, and practical utility of the n-back paradigm in both research and clinical applications (Miller et al., 2009; Koike et al., 2013; Li et al., 2019; Mencarelli et al., 2022; Mercado et al., 2022; Chen et al., 2024; Ma et al., 2024).

**TABLE 1:** n-back experiment related meta-analysis.

Theme	Number of papers and experiments	Analysis methods	Moderators tested	Effect size	Publication bias evidence	Conclusion
WM deficits in MDD with the n-back task (Nikolin et al., 2021)	31/34	Random-effects meta-analysis (SMDs) based on Acc. and RT; bias diagnostics reported when available	Age, clinical status	SMDs for Acc. (-0.23 to -0.13) and RT (0.37∼0.64) at each load level	Trim-and-fill did not change effect; no evidence of publication bias.	Patients with depression exhibit a significant decrease in accuracy on tasks of higher complexity, such as 2-back and 3-back, especially when 2-back displays the maximum effect size. Among all task complexities (including 0-back), the reaction time of patients with depression was significantly prolonged, indicating the presence of widespread psychomotor retardation.
Neuroimage for various n-back tasks (Owen et al., 2005)	24/N.A.	ALE meta-analysis	N.A.	N.A.	N.A.	The core brain regions activated by the n-back task include the dlPFC (Brodmann 46/9 area), the vlPFC (Brodmann 45/47 area), parietal cortex (including medial and lateral parietal lobes), and SMA. Verbal stimulation activates the left ventrolateral prefrontal and posterior parietal lobes more, while the nonverbal stimuli activate more of the right dlPFC and parietal lobe.
Transfer effect of n-back training (Soveri et al., 2017)	33/41	Random-effects meta-analysis (Hedges’ *g*); subgroup and moderator analyses; bias diagnostics when available	Control group type (active vs. passive)	n-back: 0.62; WM: 0.24; Gf: 0.16	PET-PEESE and funnel plot analyses show small far-transfer effect, but no strong publication bias after model correction	Explore the effects of age, training duration (dose), training type (single task vs. dual task), and task content (language vs. visual space) on transfer performance. Medium transfer effects to untrained versions of the trained n-back tasks and small transfer effects to other WM tasks, cognitive control, and Gf.
Aging and n-back performance (Bopp and Verhaeghen, 2018)	58/74	Random-effects meta-analysis (SMD) based on Acc. and RT	One level of n to the next in an n-back task, age effect at each level n	Younger: Acc.: 0.3∼0.57, RT: 0.09∼0.44 Older: Acc.: 0.33∼0.62, RT: 0.05∼0.64; Acc.: 0.28∼1.05, RT: 1.15∼1.4	Egger’s test n.s.; Funnel plot symmetric. No publication bias detected	The decline in performance of elderly people on n-back tasks is mainly related to the difficulty of focus switching, rather than overall WM load. Self-pacing allows older adults to optimize their pace and thus will enable them to compensate for age-related slowing.
Complex span and n-back measures of WM (Redick and Lindsey, 2013)	N.A.	Correlation analysis	Complex and simple span	*r* = 0.16∼0.31	No publication bias analysis reported; variability attributed to construct differences, not reporting bias	The complex span and n-back tasks cannot be used interchangeably as WM measures in research applications.
N-back WM Task for children with fMRI (Yaple and Arsalidou, 2018)	17/29	ALE meta-analysis of functional activation patterns	N.A.	N.A.	N.A.	Compared to adults, children’s consistent brain activation pattern during n-back WM tasks shows that children rely more on the posterior cortex and less on prefrontal activation. Significant concordance is observed in the insula and cerebellum for children.
The n-back task while driving (von Janczewski et al., 2021)	20/N.A.	Meta-analysis using correlation coefficients (*r*) with moderator analysis	Experimental environment, simulator fidelity, age, etc.	total effect size *r* = 0.46	Fail-safe *N* = 1,141 feffect robust, unlikely to be driven by bias	An increase in n-back levels significantly enhances cognitive load, supporting the applicability of n-back tasks as a cognitive load measurement tool in driving research.
Coordinate-based meta-analysis of the n-back WM paradigm (Wang H. et al., 2019)	96/120	ALE meta-analysis.	Age, sex, stimulus, task load, etc.	N.A.	N.A.	This work confirmed the centrality of the frontoparietal network in n-back tasks and identified key differences in activation patterns based on task conditions and participant characteristics.
fMRI evidence of age-related changes in prefrontal cortex involvement across the adult lifespan (Yaple et al., 2019)	82/N.A.	ALE meta-analysis with contrast analyses and effect-size seed-based meta-regression	Age	N.A.	N.A.	All age groups showed consistent activation in the parietal cortex and cingulate gyrus, indicating that these regions are key WM areas for n-back tasks. The insula and cerebellum also exhibit consistent activation. Prefrontal cortex engagement is concordant for young, to a lesser degree for middle-aged adults, and absent in older adults, suggesting a gradual linear decline in concordance of prefrontal cortex engagement.
Network dysfunction across psychopathologies (Farruggia et al., 2020)	N.A./160	ALE meta-analysis with contrast analyses	Task load; stimulus type	N.A.	N.A.	The psychopathologies exhibit consistent hyperactivation in the left anterior cingulate cortex/medial prefrontal cortex which is the hub region of the DMN. Abnormal activation of DMN can interfere with task-related cognitive processing, leading to a decline in WM performance.
N-back training improves Gf (Au et al., 2015)	20/N.A.	Random-effects meta-analysis (Hedges’ *g*) with subgroup and regression analyses; bias diagnostics when available.	n-back types, environment, etc.	Overall g = 0.24	Funnel plot asymmetry; far-transfer decreases to ∼0 when using active controls. → evidence of publication bias and control-design sensitivity	Through n-back training, Gf can be significantly improved, but the effect is small and is moderated by experimental design and participant characteristics.

MDD, Major depressive disorder; SMDs, Standardized mean differences; Acc., accuracy; RT, reaction time; dlPFC, dorsal lateral prefrontal cortex; vlPFC, ventrolateral prefrontal cortex; ALE, activation likelihood estimation; DMN, default mode network; SMA, supplementary movement area; Gf, fluid intelligence; bias diagnostics: Egger’s regression and Funnel plot.

Although numerous meta-analyses and reviews have examined the n-back paradigm from behavioral, neuroimaging, and training perspectives, their conclusions remain inconsistent, largely due to methodological heterogeneity (Dahlin et al., 2008a,b; Jaeggi et al., 2008, 2010b; Anguera et al., 2013; Salminen et al., 2016b; Blacker et al., 2017; Soveri et al., 2017; Flores-Gallegos and Mayer, 2022; Pahor et al., 2022). For example, studies differ in whether they treat the n-back as a measure of updating, storage capacity, or attentional control, and task implementations vary in stimulus modality, adaptive difficulty procedures, and baseline contrasts. These discrepancies contribute to well-known concerns regarding the test-retest reliability of n-back performance, the validity of training effects, and its uncertain relationship to complex span tasks, which are considered the gold standard of working memory assessment (Redick and Lindsey, 2013). Moreover, studies on neural mechanisms have reported both overlapping and divergent activation patterns across fronto-parietal and striatal networks (Jaeggi et al., 2003; Salminen et al., 2016b), which reflects unresolved debates about whether the n-back captures a unitary executive function or a composite of multiple cognitive processes. These unresolved controversies highlight a critical need for an integrated framework that not only synthesizes cognitive and neural findings across task variants but also evaluates the methodological assumptions underlying the interpretation of n-back performance. To address this gap, the present review provides a structured comparison of cognitive mechanisms, modality-specific neural recruitment, task variants, and application domains, which aims to clarify under what conditions the n-back task serves as a valid index of working memory and executive control.

Relevant studies were identified through searches in Google Scholar and PubMed using combinations of the following keywords: “n-back,” “working memory,” “updating,” “maintenance,” “attentional control,” “fMRI,” “EEG,” “training,” and “cognitive load.” Searches focused on peer-reviewed articles and meta-analyses, with no strict publication year restrictions to ensure comprehensive coverage. Additional references were identified through citation tracking of key review papers. Literature selection was guided by the thematic structure of the present review.

## 2 The n-back task: cognitive mechanisms and variants

### 2.1 Core mechanism

The n-back task primarily engages three interrelated cognitive mechanisms: updating, maintenance, and attentional control. Updating refers to the replacement of outdated information with newly relevant input. Maintenance involves the sustained activation of task-relevant representations across short time intervals. Attentional control regulates the selection of relevant items while suppressing interference from irrelevant ones. Although conceptually separable, these three components operate in parallel during n-back performance, with their relative contributions varying systematically depending on task load and stimulus characteristics. This functional interplay provides the foundation for understanding how different variants of the n-back task differentially tax executive control and neural resources.

#### 2.1.1 Updating

The defining feature of the n-back task is the continuous updating of information in working memory (WM). On each trial, participants must integrate a new stimulus while discarding the no-longer-relevant item, requiring flexible adjustment of the content and temporal order of stored representations. This dynamic updating process differentiates the n-back from static storage tasks, such as the digit span test, which emphasize capacity rather than manipulation.

Neuroimaging evidence indicates that both the dlPFC and the striatum play central roles in this updating process. Activation in the dlPFC is closely related to task load, typically showing an inverted U-shaped pattern: activity increases as WM demand rises but declines once capacity is exceeded (Callicott et al., 1999; Frank et al., 2001; Schweizer et al., 2013; Salminen et al., 2016b,c; Miró-Padilla et al., 2019). Such modulation reflects the dlPFC’s function in maintaining optimal cognitive control and updating efficiency. Consistent with these findings, our exploratory image-based meta-analysis (IBMA) using unthresholded WM maps from NeuroVault also showed robust load-dependent convergence in the bilateral dlPFC and inferior parietal lobule (IPL), aligning with the core neural architecture supporting n-back updating (see Supplementary Figures 1, 2). In contrast, striatal activation tends to increase with the need for updating, reflecting its contribution to the gating and replacement of information in WM (Schweizer et al., 2013; Salminen et al., 2016b; Miró-Padilla et al., 2019). Reduced dlPFC and IPL activation following training is often interpreted as improved neural efficiency, whereas increased striatal engagement indicates enhanced updating performance. Together, these patterns suggest that efficient n-back performance depends on a dynamic interplay between fronto-striatal circuits, where the dlPFC regulates task-load-related control demands and the striatum supports the flexible updating of memory contents.

#### 2.1.2 Maintenance

Despite its emphasis on updating, the n-back task also involves a strong maintenance component. Participants must actively retain several recent items and their temporal sequence to perform comparisons accurately. Neuroimaging studies have shown that this maintenance process depends on sustained activation within the dlPFC and parietal regions, particularly the IPL and precuneus, which together support the short-term retention and manipulation of information in WM (Braver et al., 1997; Smith and Jonides, 1998; Narayanan et al., 2005; Owen et al., 2005).

In particular, the dlPFC is thought to maintain task-relevant representations over time, enabling the comparison between current and prior stimuli, whereas the parietal cortex contributes to the temporal ordering and attentional focus necessary for accurate retrieval. Some studies also implicate the supplementary motor area (SMA) and posterior superior frontal sulcus (SFS) in sustaining sequential information, especially in visuospatial n-back paradigms (Wager and Smith, 2003; Narayanan et al., 2005).

Importantly, maintenance in the n-back task is not a passive storage process but an active one, requiring rehearsal, temporal tagging, and binding mechanisms to preserve the ordered structure of recent stimuli. The strength and stability of prefrontal-parietal coupling largely determine the upper limit of task performance as n increases, which reflects the neural capacity constraints of WM.

#### 2.1.3 Attentional control and selection

A third critical process is attentional control, which governs the selection of task-relevant information and the inhibition of irrelevant or interfering stimuli. Given the constant stream of incoming items, successful performance requires the ability to focus attention on the current trial while suppressing proactive interference from earlier ones. The parietal cortex and anterior cingulate cortex (ACC) are often implicated in this control process. The parietal cortex plays a critical role in attention allocation and spatial memory, showing robust load-dependent activation during n-back tasks (Schweizer et al., 2013; Salminen et al., 2016c; Miró-Padilla et al., 2019). The ACC is involved in error detection and stimulus monitoring, particularly in high-load or complex tasks. Post-training reductions in ACC activation, alongside changes in other cerebral regions, suggest enhanced task efficiency (Schneiders et al., 2011, 2012; Schweizer et al., 2013; Miró-Padilla et al., 2019). Notably, individual differences in attentional control, rather than memory span *per se*, have been shown to explain a substantial portion of performance variability in the n-back task (Oberauer, 2005; Kane et al., 2007; Jaeggi et al., 2010a).

Although updating, maintenance, and attentional control are often discussed as distinct components of WM, neuroimaging evidence suggests that they rely on highly overlapping neural substrates. Across n-back studies, consistent activation has been observed within a fronto-parietal network, including the dlPFC, IPL, ACC, and striatum. These regions jointly support the continuous monitoring, manipulation, and selection of information required by the task. The dlPFC serves as the central hub coordinating executive control and maintaining task goals, the IPL and precuneus contribute to short-term storage and spatial-temporal organization, the striatum facilitates flexible updating through gating mechanisms, and the ACC monitors conflicts and errors to adjust control demands.

### 2.2 n-back task variants

**FIGURE 1:**
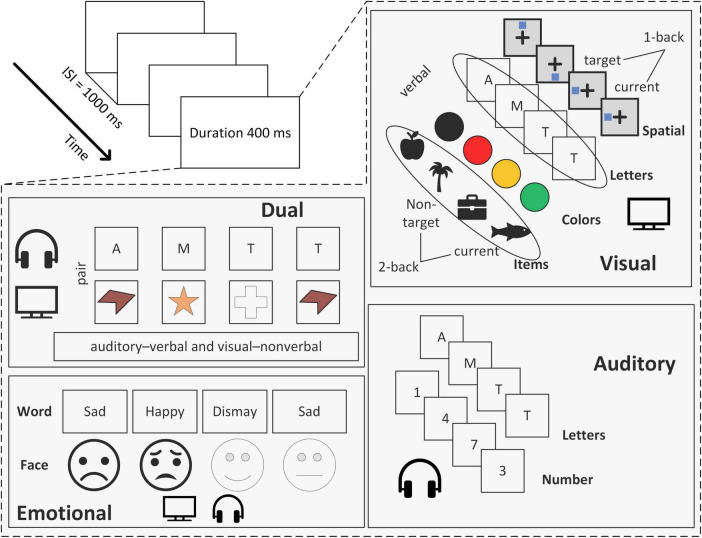
Different types of n-back tasks.

This convergence indicates that n-back performance does not reflect a single cognitive operation but rather an integrated interaction among fronto-striatal and fronto-parietal circuits. Different n-back variants can be understood as shifting the relative weighting of these three components (Figure 1). For example, increasing the value of n primarily elevates demands on updating, whereas stimulus similarity and proactive interference increase demands on attentional control. In contrast, visuospatial n-back tasks place proportionally greater demands on maintenance and parietal-mediated spatial rehearsal. Therefore, behavioral and neural outcomes across task versions can be predicted based on which component is most heavily weighted by the specific task configuration.

#### 2.2.1 Visual n-back task

The visual n-back task assesses visual working memory by requiring participants to monitor and update stimulus sequences presented in the visual modality. Common stimulus sets, such as letters, numbers, spatial markers, or objects, are typically grouped into verbal, spatial, and object-based categories (Owen et al., 2005). The choice of stimulus type is not incidental but determines the representational format and cognitive control demands of the task.

Different neural circuits are engaged depending on whether the stimuli are verbal or non-verbal. For verbal stimuli, WM involves a network that includes the ventrolateral prefrontal cortex (vlPFC), thalamus, bilateral premotor cortex, and posterior parietal cortex. In contrast, WM for non-verbal visual stimuli primarily engages the right dorsolateral prefrontal cortex (dlPFC), right medial posterior parietal cortex, and the dorsal cingulate/medial premotor cortex (Owen et al., 2005).

Behavioral and electrophysiological studies have shown that these stimulus categories differ in susceptibility to interference, response strategies, and neural activation patterns (Shalchy et al., 2020). Therefore, the visual n-back design must align stimulus selection with the specific cognitive mechanism under investigation, as varying stimulus types effectively shift the underlying neural computation supporting task performance.

#### 2.2.2 Auditory n-back task

**FIGURE 2:**
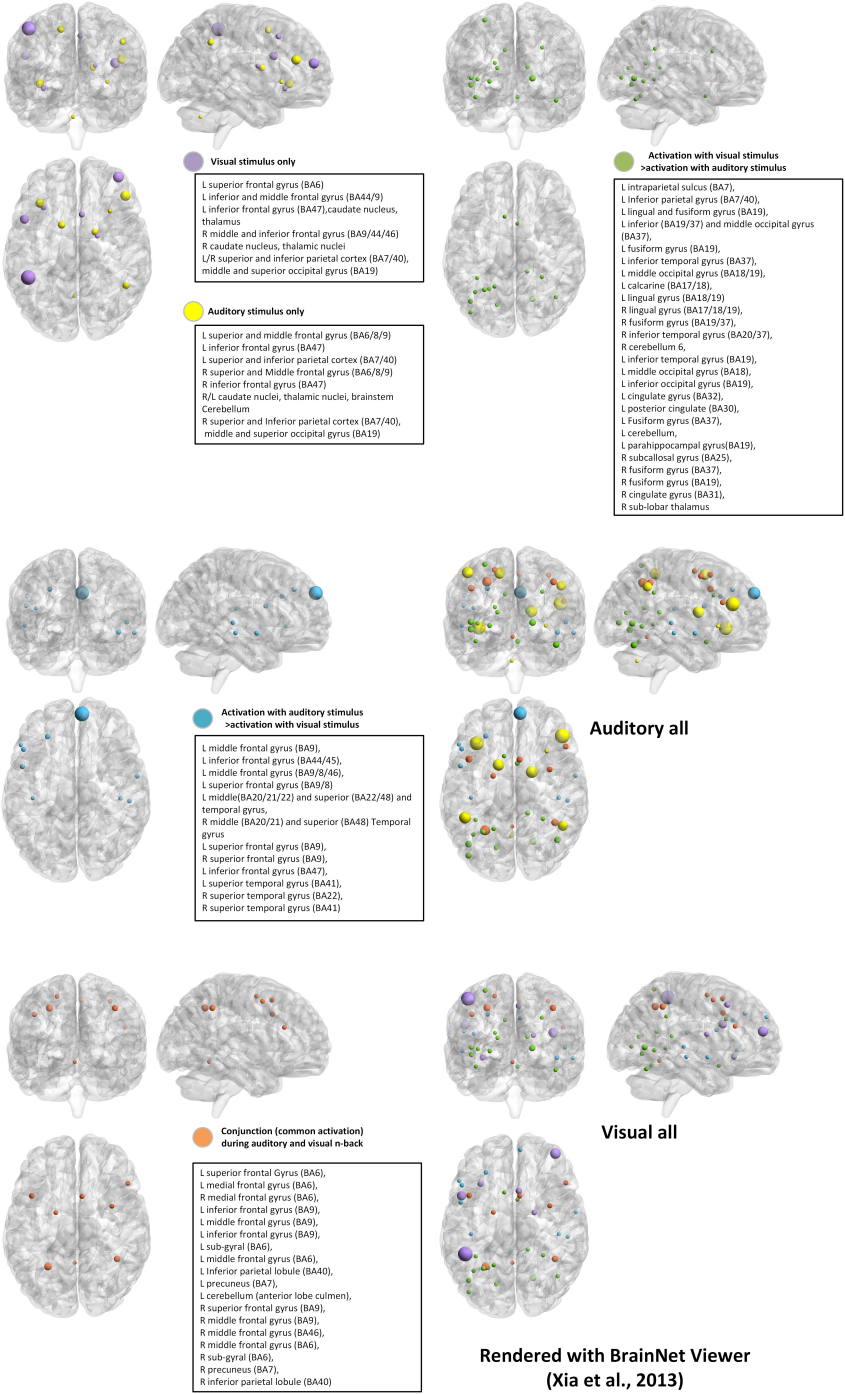
Brain activation with different stimulus modalities. We used Montreal Neurological Institute (MNI) coordinates with BrainNet Viewer (Xia et al., 2013). The BrainMesh_Ch2withCerebellum surface was adopted. The location of the nodes is derived from the peak coordinates of (Crottaz-Herbette et al., 2004; Rodriguez-Jimenez et al., 2009) and unified normalization is carried out for the size of the nodes. We plot the brain activation regions of the visual stimulus only and the auditory stimulus only in the same template (visual: purple and auditory: yellow). Then the common activation and the activation with differences for the two modalities are drawn in three templates (common: orange, auditory > visual: blue and visual > auditory: green colors). Finally, we plot brain activations for both auditory stimuli and visual stimuli, respectively.

The auditory n-back task presents sequences of spoken letters, tones, or sounds and requires updating based on auditory representations. Although stimulus modality differs, responses are typically recorded via button press rather than vocal judgments to ensure consistency in behavioral measurement (Monk et al., 2011). Relative to visual n-back tasks, auditory n-back performance is generally more accurate but slower, which reflects more stable but less rapidly accessible memory traces in the auditory domain (Allen et al., 2006; Amon and Bertenthal, 2018). In addition, there is controversy between the brain regions that support auditory n-back tasks and those that support visual n-back tasks (Figure 2; Table 2), even though both are used to study WM (Schumacher et al., 1996; Crottaz-Herbette et al., 2004; Rodriguez-Jimenez et al., 2009).

**TABLE 2:** Various types of n-back experiments with neuroimage methods.

Type	Stimuli	V. or NonV.	Load factor	Research field	Method	References	Cortical areas for related n-back task
Vis.	Number	V.	1–3	Sensitivity of ERP to WM load.	EEG	(Ren et al., 2023)	Fz, F3, F4, Pz, P3, P4, Cz, C3, C4
	Letter	V.	0–2	The function connectivity patterns of human cerebro-cerebellar circuits and their associations with verbal WM performance.	fMRI	(Li et al., 2022)	Bilateral cerebellum lobule VI, right PPC, right cuneus, right ACC, bilateral SFG
	Letter (vis. and aud.)	V.	2	Compare the pattern of brain activation while performing auditory and visual n-back tasks.	fMRI	(Rodriguez-Jimenez et al., 2009)	Bilateral frontoparietal (e.g., dlPFC), superior temporal gyrus, the ACC and occipital areas
	Spatial object	NonV.	1–4	Aging, task difficulty, and training effects on WM.	EEG	(Xu et al., 2023a)	CP1, inferior parietal lobe (IPL) (Grover et al., 2022)
	Letter	V.	0–2	Neural oscillatory processes in simple WM task	MEG	(Brookes et al., 2011)	DMN, cingulate cortex, bilateral frontal operculum, IPLs, left and right parietal lobes, left temporal lobe, left-lateralized Wernicke’s area
	Spatial object	NonV.	2	TMS treatment enhanced WM performance in a verbal digit span and a visuospatial 2-back task.	TMS	(Bagherzadeh et al., 2016)	dlPFC
	Words, pictures, and color	Both	1–3	Multi-factors like stimulus type, task structure, preprocessing method, and lab factors influence the ERP of n-back results.	EEG	(Shalchy et al., 2020)	Fz, Cz, Pz
	Number	V.	0–4	Identify characteristics of WM capacity with fMRI.	fMRI	(Callicott et al., 1999)	dlPFC, the premotor cortex, thalamus, pericingulate and superior parietal lobule
	Digits	V.	0–2	Driver workload estimation	EEG	(Lei and Roetting, 2011)	Frontal and parietal EEG spectrum
	Number	V.	0–3	auditory steady-state response and cognitive workload.	MEG	(Yokota and Naruse, 2015)	The auditory cortex, the frontal, parietal, and occipital cortices
	Number	V.	0–4	Influence of WM load to driving performance.	fNIRS	(Unni et al., 2017)	bilateral inferior frontal areas and the bilateral temporo-occipital areas
Aud.	Letter	V.	3	Verbal WM is modality-independent and is mediated by a circuit involving frontal, parietal, and cerebellar mechanisms.	PET	(Schumacher et al., 1996)	Dorsolateral frontal, Broca’s area, SMA, and premotor cortex in the left hemisphere; bilateral superior and posterior parietal cortices and anterior cingulate; and right cerebellum
	Number	V.	2	Modality effects on verbal WM with the comparison of prefrontal and parietal responses to auditory and visual stimuli.	fMRI	(Crottaz-Herbette et al., 2004)	The dlPFC, premotor and ventrolateral prefrontal cortex, intraparietal sulcus, supramarginal gyrus and the basal ganglia, the left posterior parietal cortex, the superior and middle temporal cortex and the occipital cortex
Dual	Chinese character and spatial	Both	0, 2	Brain activation of Schizophrenia patients under dual n-back tasks	fMRI	(Li et al., 2019)	The right middle frontal gyrus and the posterior parietal regions, the right hippocampus, superior parietal lobule, IPL
	Letter and shape	Both	0–3	Studies on load-dependent processing in single and dual tasks in the prefrontal cortex.	fMRI	(Jaeggi et al., 2003)	The prefrontal cortex, the precentral gyrus and the superior parietal lobule, the dlPFC
	Faces and words	Both	Ad.	Systematic WM training has the potential to augment affective cognitive control.	fMRI	(Schweizer et al., 2013)	The inferior parietal cortex, middle frontal gyrus, middle OFC, sgACC, dlPFC, IPC, hippocampus/amygdala and insula
	Letter and spatial position	Both	0, 2	Understand the cognitive and neural effects of WM training and transfer.	fMRI	(Salminen et al., 2016b)	Bilaterally in the striatum, the bilateral cuneus, the bilateral occipital cortex, the frontoparietal cortices (the bilateral premotor cortex, the bilateral PFC, and the right IPL), the right ACC, the left posterior cingulate cortex, the superior temporal lobe, the bilateral thalamus, the right middle frontal gyrus and in the left IPL
	Letter and spatial position	Both	Ad.	Dual n-back training improves functional connectivity of the right inferior frontal gyrus at rest.	fMRI	(Salminen et al., 2020)	The right inferior frontal gyrus, prefrontal cortex, the left superior parietal cortex
	Letter and spatial position	Both	Ad.	Dual n-back training produces increased integrity in white matter pathways connecting different brain regions.	MRI	(Salminen et al., 2016c)	The corticospinal tract, temporal/parietal lobes, the frontal lobe, the occipital and temporal lobes, the occipital and frontal lobes, the left and the right frontal lobes, the genuofcorpus callosum
	Letter and spatial position	Both	2	Personality traits change the impact of emotional stimuli.	Offline fNIRS	(Sugi et al., 2020)	dlPFC
Em.	Words	V.	2	Investigate the behavioral effects and neuronal correlates of emotional content and emotional components in verbal WM tasks.	fMRI	(Grimm et al., 2012)	Lateral prefrontal regions, cortical mid-line regions, dorsolateral prefrontal, the bilateral dlPFC, dACC, medial cortical regions such as the vmPFC/pACC, dmPFC, PCC, medial temporal gyrus and also in the rostral ACC and orbitofrontal cortex
	Words	V.	1	Examine the effect of stress induction on n-back performance among female students for emotional and non-emotional stimuli.	N.A.	(Khayyer et al., 2021)	Auditory cortex, hippocampus, and frontal area
	Words	V.	1–3	The influence of valence on a verbal WM task.	fNIRS	(Kopf et al., 2013)	The prefrontal cortex
	Pictures	NonV.	1, 2	Investigate interactions between WM load and affective valence with EEG.	EEG	(Grissmann et al., 2017)	Frontal and parietal area
	Face	NonV.	Ad.	ERP component P3 is highly sensitive to the influence of emotion on WM with 3-back experiments.	EEG	(Kessel et al., 2016)	The whole brain

Vis., Visual; Aud., Auditory; Em., Emotional; Ad., Adaptive; V. or NonV., Verbal or Nonverbal; PCC, posterior cingulate cortex; ACC, anterior cingulate cortex; pACC, pregenual anterior cingulate; dACC, dorsal anterior cingulate cortex; sgACC, subgenual anterior cingulate; IPC, inferior parietal cortex; vmPFC, ventromedial prefrontal cortex; dmPFC, dorsomedial prefrontal cortex; SFG, superior frontal gyrus; dlPFC, dorsolateral prefrontal cortex; DMN, default mode network; including the medial frontal cortex, medial parietal cortex, and posterior parietal lobules; SMA, supplementary motor area; OFC, orbitofrontal cortex. An adaptive (Ad.) n-back task is a dynamic variant of the n-back paradigm in which the memory load n automatically increases or decreases based on the participant’s ongoing performance. Instead of using a fixed load level (e.g., 1-back or 2-back), the task adjusts n after each block to maintain performance near an individualized difficulty threshold. Typically, n increases when accuracy is high and decreases when performance falls below a preset criterion, ensuring that the participant is consistently challenged at the upper edge of their working memory capacity (Jaeggi et al., 2008).

Importantly, the auditory n-back task is particularly suitable as a secondary workload probe because it does not interfere with ongoing visual processing and can be embedded in visually guided primary tasks to index mental workload (Zeitlin, 1995). Accordingly, auditory n-back is frequently used in driving and human-machine interaction research to assess cognitive load under real-time perceptual demands (von Janczewski et al., 2021).

#### 2.2.3 Dual n-back task

The dual n-back task simultaneously presents visual and auditory stimuli, requiring participants to monitor both modalities and update WM representations in parallel. This paradigm increases cognitive load and highlights the modality-specific nature of WM, as the neural demands of dual-task performance reflect integrated fronto-parietal and striatal engagement rather than a simple sum of single-modality n-back activations (Jaeggi et al., 2003; Salminen et al., 2016b). Because of its higher task demands, dual n-back performance has been shown to correlate with broader cognitive abilities, including individual differences in Gf and attentional control (Jaeggi et al., 2010b; Thompson et al., 2013; Li et al., 2019). The paradigm has therefore been widely used in cognitive training research. However, evidence increasingly suggests that improvements from dual n-back practice largely reflect task-specific updating efficiency, with transfer primarily observed in closely related WM tasks rather than in generalized cognitive domains (Salminen et al., 2016b).

#### 2.2.4 Modality-specific effect

The comparison between the visual and auditory all brain activation maps (Figure 2) demonstrates that sensory modality determines the entry point of information processing in n-back tasks, while higher-level WM operations are supported by a shared supramodal control network.

Specifically, visual n-back tasks reliably activate regions in the occipital cortex and dorsal parietal areas (e.g., intraparietal sulcus, superior and IPLs), reflecting reliance on visuospatial attention and visual feature updating. In contrast, auditory n-back tasks prominently recruit the superior temporal gyrus and auditory cortex, as well as inferior frontal language-related regions, consistent with phonological encoding and auditory-verbal rehearsal.

Despite these modality-dependent sensory pathways, the two tasks converge on a common fronto-parietal WM control network, including bilateral dlPFC and parietal cortex, which supports maintenance, updating, and cognitive control independent of stimulus format. This overlapping supramodal network reflects the central executive component of WM.

Furthermore, dual-modality n-back tasks require simultaneous coordination across both sensory systems, which is evident in bilateral activation expansions and engagement of additional integrative hubs, such as the temporal-parietal lobe. This network-level integration requirement suggests that dual n-back tasks impose greater executive control and cross-modal binding demands, and thus contribute to transfer effects observed in some training studies (Dahlin et al., 2008a,b; Jaeggi et al., 2008, 2010b; Anguera et al., 2013). In summary, modality determines where information enters the system (visual vs. auditory cortex), but the core working memory computations rely on a shared, supramodal fronto-parietal control network. Dual-modality conditions extend this system by imposing cross-sensory integration demands, recruiting broader and more coordinated large-scale brain networks.

#### 2.2.5 Emotional n-back task

The emotional n-back task embeds emotional valence stimuli (e.g., facial expressions or affective words) into the standard n-back paradigm to examine how affective states interact with WM (Joormann et al., 2011; Schweizer and Dalgleish, 2011; Schweizer et al., 2013; Studer-Luethi and Meier, 2021). Because emotional regulation and WM share overlapping neural substrates, particularly the dlPFC, inferior parietal cortex (IPC), and anterior cingulate cortex (ACC), emotional distraction can compete with WM for cognitive resources (Miller, 2000; Brass et al., 2005; Owen et al., 2005; Banich et al., 2009).

Neuroimaging results show that emotional valence modulates dlPFC activation during WM updating, with increased recruitment under positive or high-arousal states and reduced activation under negative states (Perlstein et al., 2002; Grimm et al., 2012; Kopf et al., 2013; Sugi et al., 2020). Emotional n-back tasks also engage affective control regions such as the subgenus anterior cingulate (sgACC), orbitofrontal cortex, amygdala, and insula, where higher WM load tends to increase frontoparietal activity while suppressing limbic responses (Schweizer et al., 2013).

Training with emotional n-back paradigms can strengthen affective cognitive control, with evidence showing increased sgACC activation and improved emotion regulation after training (Schweizer et al., 2013). Electrophysiological findings further support this interaction: negative valence decreases accuracy and slows responses, alongside reductions in theta and alpha power and alterations in P3 amplitude, reflecting increased conflict and attentional control demands (Kessel et al., 2016; Grissmann et al., 2017).

#### 2.2.6 Long-term n-back task

Long-term n-back tasks extend training across multiple sessions (typically ≥ 4 days and often ≥ 2 weeks) to examine sustained neurocognitive changes, whereas short-term n-back tasks mainly assess momentary WM performance (Aguirre et al., 2019; Miró-Padilla et al., 2019). These long-term protocols have been applied to memory improvement (Studer-Luethi and Meier, 2021), transfer effect (Jaeggi et al., 2010b; Owens et al., 2013; Heinzel et al., 2016; Lindeløv et al., 2016; Salminen et al., 2016b), aging (Heinzel et al., 2016; Salminen et al., 2016a), exploration of brain activity (Miró-Padilla et al., 2019; Nêcka et al., 2021), cognition or intelligence (Thompson et al., 2013; Ørskov et al., 2021), affective control (Schweizer et al., 2013) and rehabilitation (Aguirre et al., 2019; Jones et al., 2020; Noguchi et al., 2020).

However, long-term training outcomes show substantial individual variability, influenced by factors such as age, baseline cognitive ability, and motivation (Jaeggi et al., 2011). This suggests that long-term n-back training protocols may require adaptive or personalized implementation to optimize benefits (Ørskov et al., 2021). Additionally, because most training is conducted under controlled laboratory conditions, the ecological validity of the improvements is limited. Gamified or context-embedded designs may help better align long-term n-back training with real-world cognitive demands, particularly in children (Jaeggi et al., 2011).

### 2.3 The association between n-back tasks and other paradigms

Beyond these variants, the n-back paradigm can be better understood when viewed in relation to other working memory tasks. Comparing it with classic paradigms such as the delayed-match-to-sample (DMTS), sequential recall, and Sternberg memory tasks helps clarify which cognitive components are unique to n-back (e.g., continuous updating) and which are shared across broader memory systems.

#### 2.3.1 DMTS

The DMTS and n-back tasks are both cognitive tasks used to assess memory (Ranganath et al., 2005; Bergmann et al., 2013). In addition to commonly used stimuli such as numbers, letters, faces, etc., it also has richer stimuli such as polygons, dots, abstract design (Chen et al., 2004), object location (Bergmann et al., 2016), and ball tracking (Daniel et al., 2016). The difficulty adjustment of DMTS mainly relies on the length of delay time, whereas the variant of DMTS can increase task difficulty by sequentially increasing the number of stimuli while asking participants if the test stimuli are present in the presented stimuli (Hamilton et al., 2009), or by increasing the background of the stimuli (Bergmann et al., 2016). The delay time depends on the research content. For example, when learning novel and well-learned recognition tasks like word memory, which are relatively familiar stimuli, the delay time may be as long as 60 s (Crespo-Facorro et al., 2001). The research focuses on DMTS, and n-back tasks are different. The DMTS task primarily measures short-term memory and recognition, which assesses the ability to maintain and retrieve information over a short delay. In contrast, the n-back task measures WM updating and monitoring, requiring participants to constantly update their WM with new information and monitor for matches, making it a more dynamic task than DMTS. This experimental process results in different emphases of cognitive load for the subjects. The participants in the n-back experiment focus on attention allocation, that is, they need to be highly focused to avoid being distracted by other interfering information, while the participants in DMTS pay more attention to information integration, that is, they pay more attention to the capacity and retrieval ability of WM. Although DMTS and n-back tasks are both WM paradigms, DMTS shows some neural divergence compared to that in n-back tasks. As mentioned in the meta-analysis of DMTS in WM, DMTS does not require constant source monitoring like an n-back task does, and there is a distributed DMTS neurofunctional network consisting of 16 clusters of consistent activation (Daniel et al., 2016). The neurofunctional network of DMTS for verbal and nonverbal stimuli is quite different. For instance, the fusiform gyrus was active only in the right hemisphere in DMTS with only nonverbal stimuli, but no activation was found in n-back tasks (Owen et al., 2005). Furthermore, nonverbal stimulus creates much more brain activation in clusters located in the frontal, occipital, parietal, and limbic lobes of both hemispheres. These findings manifest that those spatial and phonological stimuli are maintained in different regions, and nonverbal stimulus sets recruit clusters from wider brain regions.

#### 2.3.2 Sequential recall task

In SR tasks, participants need to remember the order (positive or negative) of a series of objects or stimuli and recall their order or specific location after a delay (Ward et al., 2010). Similar to n-back tasks, SR tasks also need to remember the sequence of the stimuli, and the participants need to reorganize information in the short term for reverse order tasks. Such experimental stimuli are not limited to numbers. For example, the Corsi blocks tests, which are mainly used for measuring spatial WM spans. Wechsler Memory Scale III spatial span board features 10 irregularly spaced blue cubes set up on a white rectangular board, with each cube featuring an identifying number on only the researcher’s side. The researchers will touch these numbered cubes with their fingers in a predetermined order, and the participants need to recall and touch the cubes in the same order after seeing these actions. To increase WM demand by requiring the use of rehearsal, the task incorporated a 10 s delay period (retention interval) between presentation and recall (Brown, 2016). Like the n-back task, this type of task is often used to compare the impact of age on WM and focuses more on detailed behavioral performance, such as age predicting backward recall performance for both young and older adults (Brown, 2016; Xu et al., 2023a). However, the difference lies in the online updating for the n-back task with the increase in load factor, whereas SR tasks are of static memory load, which adjusts the difficulty by changing the length of the stimuli and adding the forward and backward recall for the response (Jolles et al., 2012). Through neuroimaging, it was found that the activation areas of the n-back task are mainly the prefrontal cortex (especially the dorsolateral prefrontal cortex) and parietal lobe, while the activation areas of the SR task are different, such as the hippocampus, pre-supplementary motor area, prefrontal cortex, and parietal lobe, middle temporal gyrus, and bilateral rostral anterior cingulate and inferior frontal gyri (Knutson et al., 2004; Assaf et al., 2006). The meta-analysis also validates that the bilateral SFS and the DLPFC showed the greatest specialization among frontal regions for continuous updating and temporal order memory, whereas spatial storage tasks most frequently activated the superior parietal cortex, and object storage most frequently activated the inferior temporal cortex (Wager and Smith, 2003). Since the SR task and DMTS task paradigms focus on memory and recognition, respectively, neuropsychological tests for evaluating speech learning and memory abilities have emerged, such as the California Verbal Learning Test- second edition (CVLT-II), Rey Auditory Verbal Learning Test (RAVLT), or the Wechsler Memory Scale (WMS) (Wechsler, 1945; Delis et al., 1988; Schmidt, 1996). These tests involve various experimental processes, such as various recalls and recognition tasks, and are often applied to the study of individual differences in cognitive tasks, such as the impact of age, gender, intelligence, etc., on task performance (Pelegrina et al., 2015; Whitley et al., 2016; Graves et al., 2017; Hirnstein et al., 2023). In contrast, the n-back task cannot be used for measuring individual differences due to its low reliability (Jaeggi et al., 2010a). Furthermore, the validity analysis for DMTS and SR tasks is less extensive than that for the n-back task.

#### 2.3.3 Sternberg memory task

The Sternberg memory task is a classic static WM task that contains three phases (encoding, retention, and testing). Participants are shown a set of grouped items (usually letters or numbers) that they must memorize in the encoding phase. The difficulty of the task depends on the size of the set of grouped items. Then, participants need to maintain the memorized items with WM during the delay period, with the disappearance of items in the retention phase. Finally, a probe item is shown on the screen, and participants are asked to decide whether the probe was part of the previously displayed set in the testing phase. Compared with the n-back task, the Sternberg memory task presents stimuli differently. It presents multiple stimuli simultaneously, and subjects do not need to perform cross-trial memory, resulting in a static memory load and lower experimental difficulty. Because they are both WM tasks, their brain regions also have similar performance. For example, Brookes et al. (2011) used magnetoencephalography to study the oscillations of various brain regions in different frequency bands under two WM conditions (n-back and Sternberg memory task). The frontal midline theta oscillation is closely related to WM, and there is evidence to suggest that the intensity of frontal midline theta oscillation is directly proportional to the difficulty of WM tasks (Brookes et al., 2011; Zakrzewska and Brzezicka, 2014). As the difficulty of the task increased, under the n-back experimental conditions, theta power showed more significant changes in the medial frontal cortex, indicating that the n-back experiment was more challenging. Although the n-back task and Sternberg memory task both belong to the category of WM, the brain regions recruited by the two in the experiment were not completely the same. In the Sternberg memory task, β/γ power decreases were associated with the language area (insular cortex) (Brookes et al., 2011). The delay period of the Sternberg memory task led to significant changes in the left premotor regions and Broca’s areas, which is similar to that of β/γ power decrease (Altamura et al., 2007). There is no difference in fMRI results between euthymic bipolar disorder patients and control groups at any WM load. In contrast, in the two-back task, bipolar disorder patients showed reductions in bilateral frontal, temporal, and parietal activation, and increased activations with the left precentral, right medial frontal, and left supramarginal gyri compared to control groups (Monks et al., 2004). Furthermore, researchers also investigated whether different areas of the cerebellar cortex and nuclei contribute to these two tasks (n-back and Sternberg memory task). It was shown that similar regions in the cerebellar cortex and dentate nuclei are involved in abstract and verbal n-back tasks, whereas cerebellar cortical activation was significantly stronger in the verbal version of the Sternberg memory task than in an abstract one. These findings manifest that different parts of the cerebellum seem to contribute to different aspects of WM, and right lobule VI may be more involved in verbal WM tasks (Thürling et al., 2012).

#### 2.3.4 Stroop task

In the Stroop task, participants are presented with color words like red, yellow, or blue printed in different colored inks and are asked to name the color of the ink, rather than reading the word itself. The Stroop task measures the delay in response time when naming the ink color in the incongruent condition. The delay occurs because reading is more automatic than color naming, making it challenging for the brain to suppress the impulse to read the word. Compared with the n-back task, this task evaluates cognitive control, selective attention, and response inhibition rather than WM. However, WM capacity influences the performance of the Stroop task. Subjects with higher WM capacity experience less color word interference than those with lower WM capacity (Meier and Kane, 2013). Moreover, even when lower-WM capacity subjects can respond according to goals, they take more time to resolve the interference created by each incongruent stimulus (Kane and Engle, 2003; Hutchison, 2011). In general, the Stroop task highlights attentional inhibition and conflict resolution, while the n-back task focuses more on WM updating and maintenance. The WM Stroop task, a variant of the Stroop task, increases the cognitive load by incorporating a WM component. For example, this task can be to name the color of a rectangular patch with a keypress while holding a color word in WM (Kiyonaga and Egner, 2014; Pan et al., 2019, 2022). The color patch could be congruent or incongruent with the color word being held in WM. WM, as an internally directed attention, its memory content can also affect subsequent behavior. The WM Stroop paradigm mainly distinguishes whether holding a color word in WM can produce interference in a color-discrimination task in the same manner as a color word that is perceived in the external environment (Kiyonaga and Egner, 2014). The WM Stroop paradigm is more inclined to study the guiding role of WM, while the n-back experiment focuses more on what influences WM, how to improve WM, and the relationship between WM and other cognitive abilities such as Gf (Jaeggi et al., 2010b; Chen et al., 2017; Wang et al., 2021; Xu et al., 2023a). Interestingly, the Stroop task can be combined with the n-back task (Simeonova et al., 2022). Although the Stroop-n-back paradigm allows the simultaneous manipulation of interference inhibition and working memory updating, the cognitive processes involved are highly intertwined. The task imposes concurrent demands on response inhibition, attentional control, and set maintenance, making it difficult to isolate the neural mechanisms specific to working memory. For this reason, such combined paradigms are most used in clinical or diagnostic contexts, where the objective is to maximize sensitivity to cognitive impairment, rather than to characterize the core computational or neural mechanisms of working memory itself.

#### 2.3.5 Go/no-go task

WM and response control are closely connected and integrated executive function systems (Liu et al., 2017). In the go/no-go task, participants are tested to perform an action on go stimuli and to inhibit their action for nogo stimuli. Thus, such a paradigm is used for investigating individual inhibition responses. The WM capacity also influences the inhibitory ability of an individual, as such inhibition response is related to selectively updating, maintaining, and retrieving information (Redick et al., 2011). For go/no-go tasks, the difficulty is typically adjusted by changing the ratio of go to no-go stimuli (e.g., increasing the frequency of go stimuli to make no-go stimuli less common, which makes inhibitory control more challenging). This task focuses primarily on inhibitory control with relatively low WM demands and thus, this experimental paradigm is more suitable for fine animal experiments, such as studying the changes in cognitive behavior driven by neuronal activity in specific brain regions (Pho et al., 2018; Barbosa et al., 2023). For example, through the go/no-go paradigm with different sounds and reinforcements, researchers observed how the primary auditory cortex transforms stimulus encoding from sensory representations to behavior-driven representations during task engagement, thereby specifically enhancing target stimuli across all paradigms (Bagur et al., 2018). The go/no-go task is suitable for measuring attentional processing as it requires continuous attention to detect the go stimuli, and inhibition to withhold the response for the nogo stimuli. Therefore, the go/nogo paradigm is widely used in the study of ADHD, in which the underlying dysfunction is based on the frontal-basal-ganglia-thalamo-cortical networks of the brain (Smith et al., 2004; Ogrim and Kropotov, 2019). Still, some researchers have used the n-back paradigm to study and train ADHD patients (Villemonteix et al., 2017; Jones et al., 2020). In addition, some researchers have combined these two paradigms by inserting nogo stimuli into the n-back paradigm, which requires subjects’ WM to continuously update and intermittently respond to inhibition (Breitling-Ziegler et al., 2020). This experimental paradigm, combined with ERP recording, is an economic assessment of WM and inhibition response.

### 2.4 Neuroimage for n-back tasks

Recent advancements in neuroimaging techniques have enhanced the use of n-back tasks, particularly due to the ability to manipulate WM load and systematically observe gradations in neural activity as cognitive demands increase. Key neuroimaging modalities that have been employed in conjunction with n-back tasks include fMRI (Jacola et al., 2014; Yaple et al., 2019; Assari et al., 2021), EEG (Wang X. et al., 2019; Ren et al., 2023), MEG (Brookes et al., 2011; Yokota and Naruse, 2015), PET (Schumacher et al., 1996), and functional near-infrared spectroscopy (fNIRS) (León-Domínguez et al., 2015; Chen et al., 2024). Table 2 provides a summary of n-back experiments utilizing different stimuli and neuroimaging methods.

**FIGURE 3:**
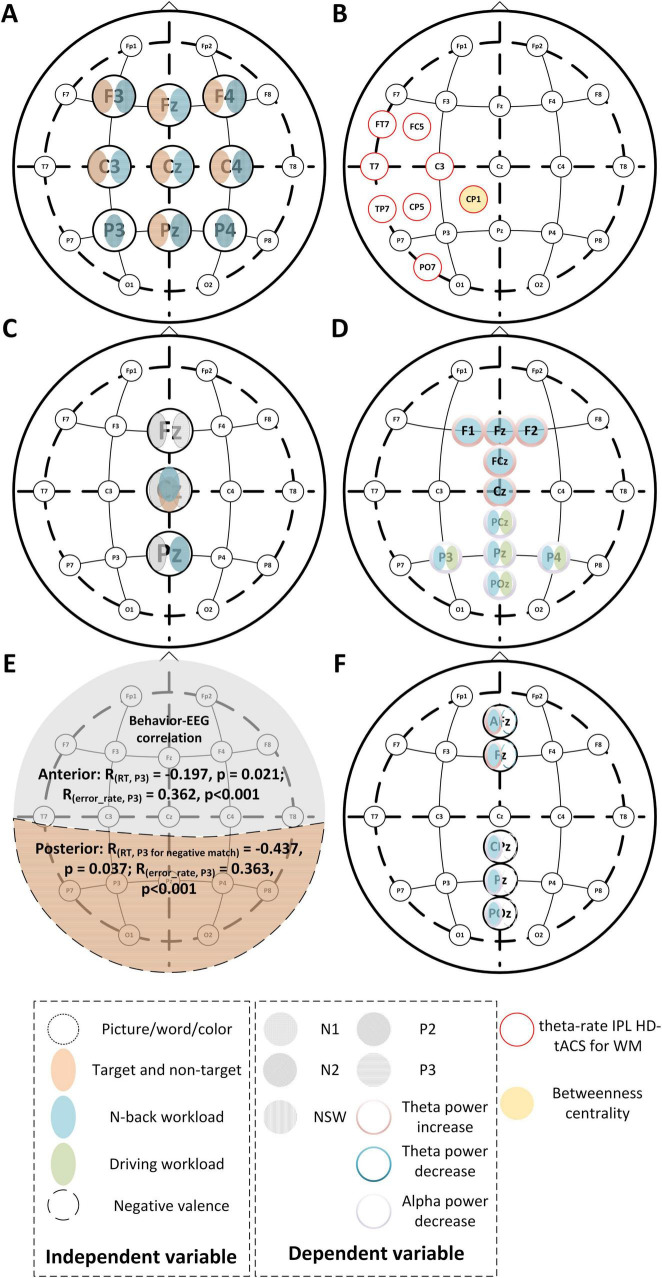
Representative EEG changes for different n-back tasks. (**A,C–F**) Different independent variables lead to brain regional changes of time domain and frequency domain features like N1, P3, and theta power, etc. The correspondence between the subfigure and literature is **(A)** for Ren et al. (2023), **(C)** for Shalchy et al. (2020), **(D)** for Lei and Roetting (2011), **(E)** for Grissmann et al. (2017), **(F)** for Kessel et al. (2016). **(B)** The inferior parietal lobe where applying transcranial alternating current stimulation (tACS) can improve verbal WM (Grover et al., 2022). The CP1 region is not only the stimulation area of transcranial alternating current but also the prominent point of betweenness centrality in the process of brain network construction in spatial n-back experiments (Xu et al., 2023a).

EEG-based analyses of n-back tasks can be approached from both the time-domain (event-related potentials, ERPs) and frequency-domain perspectives (theta, alpha, beta, and gamma bands). Figure 3 highlights typical EEG changes during the n-back process under varying stimuli and cognitive loads. EEG signals, with their high temporal resolution, are particularly effective in capturing rapid changes in cognitive load, such as transitions from 1-back to 3-back (Brouwer et al., 2012; Wang et al., 2016; Ren et al., 2023). In addition, EEG is a valuable electrophysiological marker of cognitive workload. For example, a decrease in alpha-band power in the parieto-occipital region indicates increased cognitive load, while an increase in theta-band power in the prefrontal cortex reflects heightened attentional demands (Brouwer et al., 2012; Roy et al., 2013; Puma et al., 2018; Maimon et al., 2020; Ren et al., 2023; Figure 3). Thus, EEG can serve as an effective tool for detecting cognitive workload in n-back tasks (Brouwer et al., 2012; Wang et al., 2016; Grissmann et al., 2017; Hamann and Carstengerdes, 2022).

Furthermore, ERPs, due to their component specificity and sensitivity to task parameters, provide insights into task-related cognitive processes. Components such as P2, N2, P3, and negative slow wave (NSW) are commonly studied to understand various cognitive functions involved in the n-back paradigm. For instance, P2 is associated with early sensory processing, N2 with inhibitory control, and P3 with cognitive updating (Chen et al., 2008; Kessel et al., 2016; Grissmann et al., 2017; Shalchy et al., 2020; Ren et al., 2023). Notably, P3 is influenced by both the trial type (target vs non-target) and input valence, with the amplitude of P3 significantly increasing in the posterior regions (Kessel et al., 2016; Shalchy et al., 2020; Ren et al., 2023).

Finally, EEG can bridge the gap between behavioral and electrophysiological measures, as changes in ERP components often correlate with task performance. For example, poorer performance on higher-load n-back trials is typically reflected in altered ERP patterns (Kessel et al., 2016; Shalchy et al., 2020).

**FIGURE 4:**
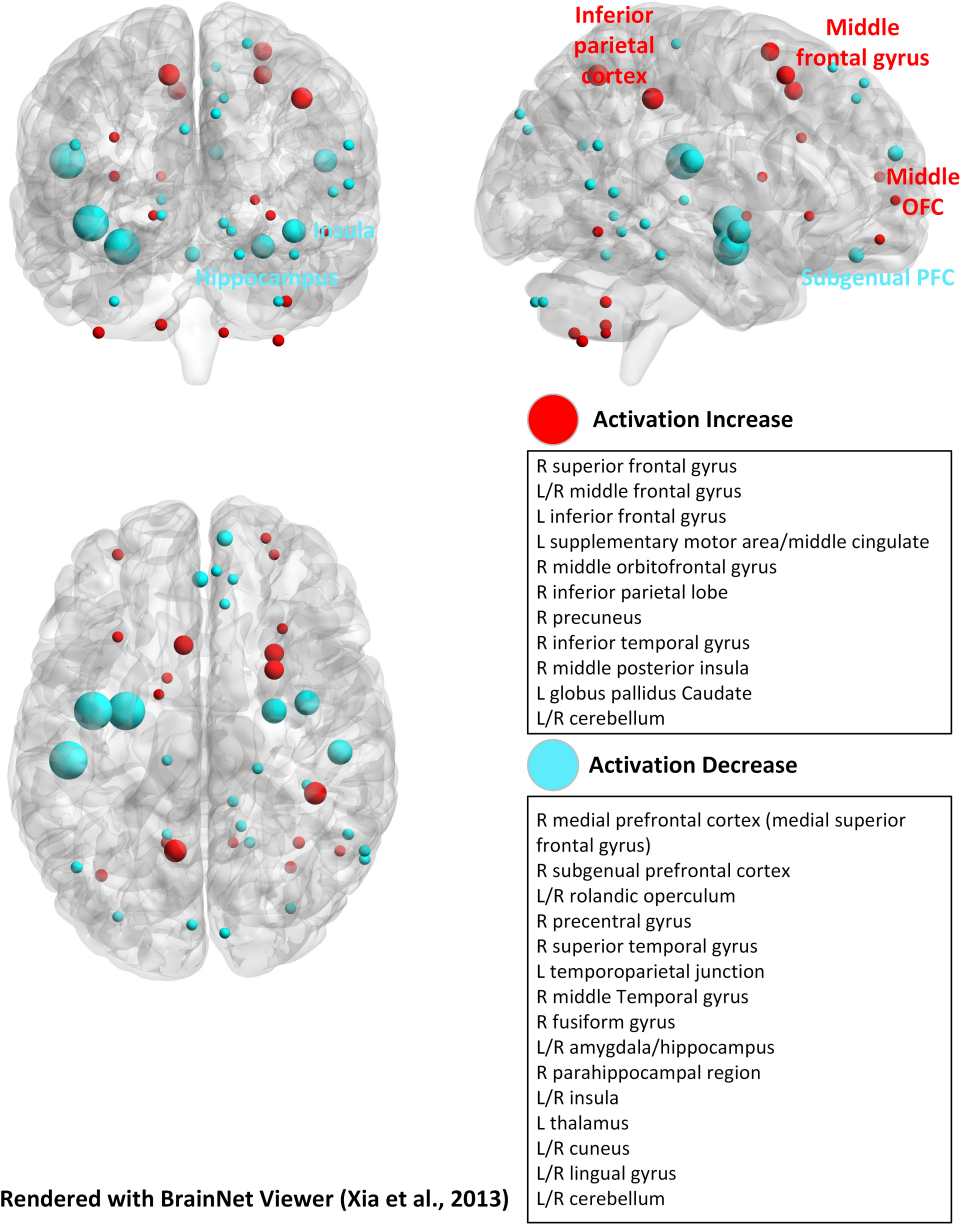
Brain activation regions for the emotional dual n-back task (Schweizer et al., 2013). The brain template and the plot method are the same as in Figure 2.

The majority of experimental analyses in n-back tasks come from fMRI, primarily due to its high spatial resolution, which allows precise localization of brain activation patterns. This capability is critical for studying the distributed networks involved in WM tasks. For example, different modalities of stimulus boost different brain regional activation, whereas the emotional dual n-back task promotes the increase or decrease of activation in different sites (Callicott et al., 1999; Crottaz-Herbette et al., 2004; Rodriguez-Jimenez et al., 2009; Schweizer et al., 2013; Simeonova et al., 2022). Due to differences in activation of visual n-back and auditory n-back brain regions, as well as differences in application areas, we redrew activation maps of brain regions in the n-back experiment based on these two stimulus modalities (Crottaz-Herbette et al., 2004; Rodriguez-Jimenez et al., 2009; Figure 2). It is shown that the cerebellum, which contributes to motor learning, also engages in the dual n-back task (Salminen et al., 2016b; Li et al., 2019). We also summarized the activation of brain regions during the emotional dual n-back process, aiming to help us better understand the relationship between emotionally related brain regions and WM updating (Schweizer et al., 2013; Figure 4). Additionally, fMRI intuitively tracks changes in brain activity as WM load increases, providing insights into the transition from simple to complex multimodal tasks (Callicott et al., 1999; Salminen et al., 2016c). For instance, in a standard n-back task, frontal activation tends to decline when processing demands become excessive. However, Jaeggi et al. (2003) observed an increase in prefrontal activation during dual n-back tasks, even when processing demands were at their highest, particularly in the most difficult conditions. This suggests that fMRI is an excellent tool for investigating both training effects and long-term changes associated with n-back tasks. Unlike EEG-based analysis, which primarily focuses on frequency bands and rough regional divisions, fMRI provides a more detailed examination of brain regional activation during n-back tasks.

Neuroimaging for n-back experiments may also use fNIRS devices, mainly due to their portable performance and better spatial resolution than EEG. For example, in the verbal WM task, increased complexity needs greater executive control, thus leading to an increase in cerebral blood flow to the areas associated with verbal WM (León-Domínguez et al., 2015).

Overall, different neuroimaging modalities provide complementary insights into the neural mechanisms engaged during n-back performance. EEG-based n-back paradigms are typically conducted online, allowing millisecond-level tracking of rapid updating, attentional switching, and workload fluctuations. However, their spatial resolution is limited. In contrast, fMRI studies fall into two major designs: (1) pre-post training structural or resting-state scans (Salminen et al., 2016c,^2020^), which capture longer-term plasticity but do not reflect real-time updating dynamics, and (2) online task-fMRI designs (Li et al., 2019), which compare activation patterns across difficulty levels to localize fronto-parietal engagement, but remain constrained by the slow hemodynamic response and therefore cannot resolve the fine-grained temporal sequence of cognitive operations. Thus, even when fMRI is collected during task execution, it primarily reflects load-dependent activation differences, rather than the moment-by-moment updating processes that define the n-back paradigm.

Future research should therefore prioritize multimodal integration, especially approaches that combine EEG’s high temporal precision with fMRI’s high spatial specificity to construct dynamic functional network models of WM updating. Such joint acquisition and source-constrained analysis pipelines would allow researchers to map how neural representations evolve across stimulus encoding, delay, comparison, and response stages, an aspect that cannot be fully characterized by either modality alone. These multimodal frameworks are particularly critical for understanding n-back paradigms involving emotional or dual-task components, where multiple cognitive processes interact over short timescales. Accordingly, advancing n-back neuroimaging requires shifting from static localization toward temporally resolved network dynamics, which can leverage the strengths of both EEG and fMRI in a coordinated analytical framework.

### 2.5 Stimulation effect (tDCS and tACS) on n-back tasks

Non-invasive electrical stimulation studies using tDCS and tACS show that the effects on n-back WM performance are strongly condition-dependent rather than uniformly facilitatory (Emmert et al., 2016). Meta-analytic evidence indicates that single-session tDCS produces inconsistent or small behavioral effects, whereas multi-session stimulation combined with WM training is more likely to yield improvements, typically reflected in reduced reaction time rather than accuracy gains (Senkowski et al., 2022). Stimulation of the left dlPFC remains the most common approach, and its effects are supported by neural markers such as enhanced P3 amplitude and stronger task-related default-mode network suppression, which correlate with faster responding (Abellaneda-Pérez et al., 2020; Dong et al., 2020). In contrast, tACS exhibits more robust single-session effects, particularly when theta-band stimulation is applied across fronto-parietal networks to enhance phase synchronization, supporting updating and executive control during n-back performance (Pahor and Jaušovec, 2018). A recent meta-analysis confirms that theta tACS reliably improves cognitive performance in healthy adults, with stronger effects when stimulation is delivered online during task execution (Lee et al., 2023). Gamma-band tACS and cross-band tACS, on the other hand, often produce neural entrainment without consistent behavioral improvement, suggesting differential roles for frequency-specific circuit modulation (Pahor and Jaušovec, 2018; Diedrich et al., 2025).

Moreover, studies targeting the right dlPFC and parietal cortex highlight that WM performance is not exclusively governed by left prefrontal regions. Parietal stimulation may more robustly enhance spatial working memory, particularly under offline protocols (Živanović et al., 2021). Finally, repetitive HD-tACS demonstrates that frequency-region combinations can selectively and durably enhance distinct memory systems, producing double dissociation between WM and LTM and effects lasting up to 1 month (Grover et al., 2022). These findings collectively support the view that network-level targeting and stimulation timing are critical determinants of cognitive outcomes, rather than stimulation modality alone (Table 3).

**TABLE 3:** tDCS/tACS effects on n-back task.

Stimulation type	Typical target region/montage	Neural mechanism	WM subprocesses most affected (n-back)	Task timing (online/offline)	Typical behavioral effects	Network/neural effects	References
tDCS (left dlPFC)	F3 (standard bipolar) or HD-tDCS	Increased cortical excitability; facilitates prefrontal control	Updating and maintenance	Multi-session online or combined training	Single-session effects inconsistent; multi-session often reduces RT	Increased P3 amplitude; stronger task-related DMN suppression	(Abellaneda-Pérez et al., 2020; Dong et al., 2020; Senkowski et al., 2022)
tDCS (right dlPFC/PPC)	F4 or PPC	Attention allocation and fronto-parietal resource rebalancing	Spatial updating and visuospatial WM	Mostly offline	More consistent improvements in 3-back and spatial WM	Highlights functional relevance of parietal WM nodes	(Živanović et al., 2021)
tDCS (broad-frontal)	F3/F4 with extracephalic return	Broad prefrontal excitability shift	Updating under moderate load	Online	Improved 2-back RT selectively	Montage strongly determines effect direction	(Martin et al., 2023)
tACS (theta 4–7 Hz)	Fronto-parietal dual-site	Synchronizes fronto-parietal network rhythms	Executive control and updating	Online	More reliable accuracy improvements vs. single-session tDCS	Increased theta coherence and task-phase coupling	(Pahor and Jaušovec, 2018; Lee et al., 2023)
tACS (gamma 30–80 Hz)	dlPFC or PPC	Enhances fast binding /activation cycles	Short-term maintenance	Online	Behavioral effects variable	Strong oscillatory entrainment without consistent accuracy gains	(Pahor and Jaušovec, 2018)
HD-tACS (rhythm-specific, older adults)	IPL-θ vs. dlPFC-γ	Frequency-region dissociation in memory systems	WM (θ) vs. LTM (γ)	4-day multi-session	Double dissociation maintained ≥ 1 month	Long-term plasticity in WM/LTM circuits	(Grover et al., 2022)
Cross-frequency theta–gamma coupled	dlPFC	Peak-locked θ–γ coupling to enhance hierarchical control loops	High-load updating (2-back > 1-back)	16-session multi-week training	Improved 2-back discriminability (sensitivity and decision criterion); no effect on 1-back	Behavioral gains accumulated across training weeks	(Diedrich et al., 2025)

tDCS, transcranial direct current stimulation; tACS, transcranial alternating current stimulation; HD-tDCS, high-definition transcranial direct current stimulation; PPC, posterior parietal cortex; dlPFC, dorsolateral prefrontal cortex; IPL, inferior parietal lobule; DMN, default mode network; RT, reaction time; LTM, long-term memory.

## 3 Applications of n-back experiments

### 3.1 Cognitive training and transfer effects

Cognitive training needs to consider the transfer effect (TE) that improves cognitive functions through specialized training and examines the extent to which these improvements transfer to untrained tasks or real-world scenarios. In cognitive training, both standard experimental paradigms, such as letter memory, dual n-back tasks, and cognitive tasks hidden in games, may produce a certain degree of TE (Dahlin et al., 2008a,b; Jaeggi et al., 2008, 2010b; Anguera et al., 2013; Flores-Gallegos and Mayer, 2022; Pahor et al., 2022). However, the TE is often related to the age of the participants and the correlation between the training task and the transfer task (Dahlin et al., 2008a,b; Li et al., 2008; Borella et al., 2010, 2013; Anguera et al., 2013). The TE can be categorized into near transfer and far transfer according to the relevance between the training activity and the skills to be improved. Usually, generating a near transfer indicates that the untrained task relies more on WM, while generating a far transfer means that the untrained task relies on other cognitive functions such as attention, reasoning, etc. (Klingberg, 2010; Pergher et al., 2018). The TE of cognition needs to be confirmed through corresponding cognitive tests, such as the delayed-recognition WM task for TE of WM and the test of variables of attention for sustained attention after cognitive training (Clapp et al., 2011; Clapp and Gazzaley, 2012; Anguera et al., 2013; Leark et al., 2024). The TE of the n-back experiment involves a wide range of cognitive functions, such as spatial memory, attention, and Gf (Heinzel et al., 2014a,b; Pergher et al., 2018). Gf refers to the ability to reason, solve novel problems, and adapt to new situations independently of previously acquired knowledge or experience. Since the Gf relies on WM and both Gf and WM share variance like underlying neural circuitries (Bouker and Scarlatos, 2013), and the n-back experiment is considered to be able to test and improve WM, the n-back experiment is believed to have a positive transfer effect on Gf (Kane and Engle, 2002; Gray et al., 2003). The TE between n-back training and Gf was first systematically introduced by Jaeggi et al. (Jaeggi et al., 2008). A dual n-back task that combined visual and auditory stimuli was given with an adaptive training strategy.

The pretesting and post-testing were provided for a measure of Gf with either the Raven’s Advanced Progressive Matrices (RAPM) test or the short version of the Bochumer Matrizen-Test (BOMAT) for different groups (Horn, 1962; Raven, 1962; Hossiep et al., 2001; Heinzel et al., 2016). Such TE to Gf was observed in both young and elderly groups with various neuroimaging methods (Heinzel et al., 2014a,^2016^; Pergher et al., 2018). The meta-analysis of 20 studies also supports this conclusion that the effect size of the treatment/control group difference in Gf at posttest vs. baseline of Hedge’s *g* at 0.24 vs. -0.003 (Au et al., 2015). However, some studies have also found that the n-back experiment is not very significant for the TE of Gf (Thompson et al., 2013; Melby-Lervåg and Hulme, 2016; Melby-Lervåg et al., 2016; Blacker et al., 2017; Ripp et al., 2022; Juras et al., 2024). A multi-level meta-analysis including 33 studies focused on the TE of the n-back task to the untrained n-back task and other cognitive processes (Soveri et al., 2017). The TE of n-back to Gf [*g* = 0.16, (0.08, 0.24), *p* < 0.001] is in line with that in (Au et al., 2015). However, the TE is much smaller than the untrained n-back task [*g* = 0.63, (0.44, 0.82), *p* < 0.001), which manifests that the transfer following WM training with the n-back task is a task-specific one instead of a widespread TE (Salminen et al., 2016b; Blacker et al., 2017; Soveri et al., 2017). Further research suggests that the TE of dual n-back (containing visual-spatial components) may not depend on relational WM, which is highly correlated with the spatial n-back task, but rather on other mechanisms (Blacker et al., 2017).

### 3.2 Clinical applications

The n-back task is widely used in clinical settings to evaluate WM and related executive functions, demonstrating good sensitivity to WM load and neural engagement (Kearney-Ramos et al., 2014). Its clinical application spans diverse conditions beyond neurodegenerative diseases such as Alzheimer’s and Parkinson’s disease, including psychiatric disorders (e.g., schizophrenia), chronic pain, and spinal cord injury, where deviations in n-back performance and cortical activation patterns reflect impaired WM processing (Miller et al., 2009; Koike et al., 2013; Li et al., 2019; Mencarelli et al., 2022; Mercado et al., 2022; Chen et al., 2024; Ma et al., 2024). Owing to its compatibility with neuroimaging and neurostimulation techniques such as fMRI, fNIRS, MEG, and rTMS, the n-back task has also been used to assess how measurement environments and neural modulation influence task behavior and brain activation patterns (Ciesielski et al., 2010; Gaudeau-Bosma et al., 2013; Herff et al., 2014; Jacola et al., 2014; León-Domínguez et al., 2015; Simeonova et al., 2022). For instance, reaction times are generally slower during fMRI scanning, and rTMS stimulation of the left dlPFC can alter activity in broader WM-related networks during n-back performance (Gaudeau-Bosma et al., 2013; Jacola et al., 2014).

In addition to behavioral performance indices, computational and electrophysiological analyses of n-back behavior contribute to deeper cognitive and neurobiological interpretation. For example, drift-diffusion modeling has been used to relate n-back decision dynamics to genetic risk markers in youth populations, linking WM efficiency to polygenic vulnerability signatures (Pedersen et al., 2023).

Beyond assessment, the n-back task is also used as a rehabilitation tool. Training studies in traumatic brain injury (TBI), multiple sclerosis (MS), and stroke populations have shown that repeated n-back practice can improve WM task performance and enhance neural efficiency in frontoparietal networks (Vallat-Azouvi et al., 2009, 2014; Lindeløv et al., 2016; Aguirre et al., 2019; Gimbel et al., 2021). Similarly, improvements have been reported in hemodialysis patients and in children with ADHD following n-back-based cognitive training (Jones et al., 2020; Noguchi et al., 2020). However, these gains largely reflect near transfer, meaning improvements are typically limited to tasks sharing similar WM updating demands. For example, in children with ADHD, n-back training improved the trained task and yielded small-to-moderate gains in closely related inhibitory control measures (e.g., untrained n-back and inhibitory control, η^2^_p_ = 0.13 at post-test), but did not produce reliable far-transfer effects on broader executive or academic outcomes, and some improvements diminished at follow-up (Jones et al., 2020).

Age-related cognitive changes further shape the clinical application of the n-back task. With aging, individuals shift from interference-resistant executive control strategies toward greater reliance on attentional and mnemonic support processes during n-back performance (Gajewski et al., 2018; Yaple et al., 2019). This shift aligns with broader compensatory recruitment patterns described in aging, mild cognitive impairment (MCI), and Alzheimer’s disease (Reuter-Lorenz and Park, 2014; Kirova et al., 2015). Correspondingly, electrophysiological features derived from n-back tasks, such as ERD/ERS and ERP components, have shown potential in early differentiation of healthy aging, MCI, and mild Alzheimer’s disease (Fraga et al., 2017, 2018; Yaple et al., 2019). Despite this diagnostic value, n-back training is not commonly adopted as a rehabilitation intervention for MCI or Alzheimer’s disease, likely due to the abstract nature and difficulty of the task, as well as evidence that cognitive training in these populations is more effective when integrated into ecologically relevant daily activities rather than isolated WM tasks (Barnes et al., 2009; Li et al., 2011; Kirova et al., 2015).

While many studies report positive effects, several methodological constraints require caution: (1) sample sizes are often small (*n* < 30 in many trials), (2) training duration and intensity vary widely across studies, (3) outcome metrics differ across behavioral and neural endpoints, complicating effect estimation, and (4) placebo and active-control conditions are sometimes insufficient, particularly in home-based training protocols. Therefore, current evidence supports n-back as a clinically useful evaluation tool and as a targeted rehabilitation method in conditions such as TBI and MS. However, it should not be considered a universal cognitive training protocol, particularly in ADHD and MCI populations, where far-transfer effects remain unreliable.

### 3.3 Children and education

Unlike n-back, which can be influenced by familiarity-based strategies, recall-based WM tasks require active maintenance and manipulation, making them developmentally appropriate and behaviorally sensitive training tools for children. (Roughan and Hadwin, 2011; Alloway et al., 2013).

This does not mean that the n-back experiment is unimportant, on the contrary, the n-back task plays an important role in the education of children and adolescents, mainly manifested in the discrimination of neural and cognitive growth trajectories (Ciesielski et al., 2006; López-Vicente et al., 2016; Moisala et al., 2017; Chaarani et al., 2022), data collection and children’s diseases exploration (Pelegrina et al., 2015; Yaple and Arsalidou, 2018; Jones et al., 2020), the influence of family economic status on children’s cognition (Assari et al., 2020, 2021; Murtha et al., 2022), intelligence and neuropsychological development assessment (Schleepen and Jonkman, 2009; Forns et al., 2014; Ariës et al., 2015; Tayeri et al., 2016; Khayyer et al., 2021; Ørskov et al., 2021; Zhong et al., 2024), development of educational tools (Liang and Fu, 2024), research on special children’s education (Yanai and Maekawa, 2011). There are two main types of n-back experiments related to adolescents and education. One type uses n-back experiments for testing, such as testing WM and other factors that affect WM, such as volleyball, games, etc. (Verhaeghen and Basak, 2005; Schleepen and Jonkman, 2009; Yanai and Maekawa, 2011; Moisala et al., 2017; Chaarani et al., 2022; Ebert et al., 2024). The test results of n-back can also reflect some social issues, such as the education level of parents and family income status (Schleepen and Jonkman, 2009; Assari et al., 2020; Murtha et al., 2022). Another type of n-back experiment mainly studies the function of n-back on adolescents, such as its impact on growth trajectory or the improvement of reasoning ability (Ciesielski et al., 2006; Pelegrina et al., 2015; López-Vicente et al., 2016; Tayeri et al., 2016; Johann and Karbach, 2018; Jones et al., 2020; Gómez et al., 2024). Sustained selective attention and WM are both crucial cognitive functions for children and adolescents.

However, interventions to enhance sustained attention for children and adolescents are usually continuous performance tasks (CPT) such as go/no-go tasks, although n-back cognitive training is believed to require sustained attention and has also been used for training tasks in children with ADHD (Verhaeghen and Basak, 2005; Jones et al., 2020; Slattery et al., 2022). This is because the n-back task beyond the 1-back level may be difficult for young children (Pelegrina et al., 2015; Ebert et al., 2024). WM ability is closely related to various learning abilities, such as reasoning skills. Combining cognitive WM capacity training (n-back tasks) and reasoning strategy training can improve reasoning skills in history courses (Ariës et al., 2015). In contrast, merely cognitive WM capacity training has less impact on improving school students’ reasoning skills. Such a result manifests that the combined training strategy facilitates the internalized reasoning structure in the WM (Ariës et al., 2015).

The n-back tasks related to children’s education are usually made more gamified (Jaeggi et al., 2011; Bouker and Scarlatos, 2013; Johann and Karbach, 2018; Bernecker and Ninaus, 2021). The gamified version of n-back tasks leads to higher engagement and self-reported motivation, and some scholars reported that there is no performance difference from that in the standard version (Johann and Karbach, 2018).

### 3.4 Social sciences and the n-back task

The relationship between the WM and social cognition is the focus of discussions in the social sciences and n-back tasks. Social cognition involves the recognition of others as well as the recognition of oneself. As far as understanding oneself is concerned, the n-back task, together with other tasks like the Tower of Hanoi task, Stroop test, and Wisconsin card sorting test, is used for self-regulation empowerment training, which can improve the neurocognitive and social skills in students with dyscalculia (Rahbar Karbasdehi et al., 2019). Improving social cognition requires regulating emotions well. As mentioned before, n-back tasks often introduce emotional dimensions, but the research content usually focuses on the impact of emotions on WM or attentional control, such as positive emotions can prolong attention span in WM (Bartolic et al., 1999; Villemonteix et al., 2017; Dong-ni et al., 2018; O’Brien et al., 2020; Ra̧czy and Orzechowski, 2021). Several studies have confirmed that n-back training has a positive effect on affective control (Gross, 2002; Schweizer et al., 2011, 2013). There is a close connection between language and social sciences. Monolingual and bilingual young people have different results in emotional n-back experiments, such as differences in accuracy and the influence of emotional stimuli (Barker and Bialystok, 2019). An important aspect of social cognition is perspective-taking, and research has shown that social WM training improves perspective-taking accuracy. However, social WM training is not like an n-back task, it is more like an SR task. Participants need to rank friends along with trait dimensions in WM. Another experiment of perspective taking involves moving items on the bookshelf based on voice prompts, which is somewhat similar to the 1-back experiment. However, the difference is that the speaker is behind the bookshelf, while the observer (subject) is in front of the bookshelf, and a part of the back of the bookshelf is covered by a board. This requires empathy to determine whether the object being moved is what the speaker wants to move (Meyer and Lieberman, 2016).

The impact of social pressure on individuals can also be achieved through n-back tasks. For example, cortisol responses provide an acute stress environment and higher cortisol responders for young people show better performance on n-back tasks (Lin et al., 2020). The generation of stress does not necessarily require the action of medication but rather involves informing participants of their performance and comparison with others during the n-back process (Smeding et al., 2015; Wang X. et al., 2019). The social pressure that people feel may stem from prolonged exposure to socio-economic hardening. Poverty-related cognitive and emotional stress may exacerbate neurocognitive function and lead to impulsive delayed reward discounting and emotional reactivity is closely related to delayed reward discounting. In emerging adults with high emotional reactivity, the severity of socio-economic hardship indicates an increase in delayed reward discounting, which is achieved through a decrease in brain region responses activated during n-back WM tasks (Oshri et al., 2019). In addition, the n-back experiments applied in social sciences also include the impact of family status on adolescents, such as household income, parental education level, and race (Akhlaghipour and Assari, 2020; Assari et al., 2020, 2021; Lurie et al., 2024).

## 4 Limitations and challenges of n-back tasks

### 4.1 N-back experiment and mental fatigue

Mental fatigue is a critical confounding factor in n-back research because it directly influences both behavioral performance and neural activation patterns. Prolonged cognitive load or sustained task engagement can lead to reduced accuracy, slower reaction times, and altered ERP and fMRI signals, making it difficult to determine whether observed effects reflect WM processes or the impact of fatigue.

However, despite its importance, mental fatigue is notoriously difficult to measure objectively. For instance, feelings of tiredness or reduced alertness are easily confounded with boredom (Pattyn et al., 2008; Smith et al., 2019; Hassan et al., 2024). When the experimenter conducts a driving fatigue test on the simulation platform, it is difficult to objectively judge whether the subject is in a state of fatigue or boredom during the brief 1-h driving process (Xu et al., 2023b). In many experiments studying mental fatigue, it is necessary to introduce a state of mental fatigue, and the n-back task is one of the means (Shortz et al., 2015; Argüero Fonseca et al., 2023). N-back has been proven to be an acute way of introducing fatigue, typically taking only half an hour to an hour (Clark et al., 2019; Argüero Fonseca et al., 2023). The increase in difficulty of the n-back experiment has raised the cognitive demands for the task, making it easier to enter a state of fatigue, resulting in a decrease in the amplitude of the electroencephalogram (P3a) in the Fz, Cz, and Pz regions (Massar et al., 2010). However, experiments or training based on n-back rarely mention the issue of mental fatigue during the experimental process, which may be due to the following reasons. Firstly, the induce of mental fatigue state varies from person to person, even for individuals with similar traits such as age, disease, etc., especially when facing high WM load tasks, there may be significant differences in tolerance and fatigue performance (Wylie et al., 2017; Pergher et al., 2019; Morris et al., 2024). Secondly, for the induce of fatigue state, different experimental paradigms vary greatly (Bray et al., 2011; Budini et al., 2014; Hassan et al., 2024). Due to the experimental setup, such as the gamification setting, the duration of the n-back experiment may not induce fatigue to most participants (Jaeggi et al., 2011; Johann and Karbach, 2018). Multi-sensory input (dual n-back tasks) may mobilize more brain resources, thereby reducing fatigue during the experimental process (Jaeggi et al., 2008, 2010b; Salminen et al., 2020). Meanwhile, adding rest time in the experiment can avoid the occurrence of fatigue (Herff et al., 2014). Thirdly, many n-back tasks require continuous training over several days, so repeated training can help participants better adapt to the experimental process and ignore the effects of fatigue (Jaeggi et al., 2008, 2010b; Thompson et al., 2013; Salminen et al., 2016a). Finally, measuring mental fatigue is relatively difficult, as it typically requires additional physiological or behavioral indicators to evaluate, such as decreased task performance, subjective fatigue reports, or physiological responses (such as skin conductance, heart rate variability, blink frequency, etc.). Moreover, the data to be observed in experiments is easily affected by fatigue, like accuracy and reaction time. Meanwhile, controlling for too many variables can affect the interpretability of research results. Therefore, sometimes the impact of mental fatigue is directly ignored.

To mitigate these effects, future studies should adopt more systematic approaches to monitor and control fatigue. First, experimenters can use adaptive task designs that automatically adjust difficulty based on performance or physiological indicators, thereby maintaining engagement without excessive strain. However, current adaptive task designs mainly consider the task difficulty for participants rather than the influence of mental fatigue (load factor in Table 2). Second, rest intervals or shorter task blocks should be incorporated to minimize cumulative fatigue, particularly in long or multi-session paradigms. Third, integrating objective physiological measures, such as electrodermal activity, heart rate variability, or ocular metrics (blink rate measure, pupillometry), can help detect early signs of mental fatigue and distinguish them from boredom or disengagement. In addition, self-report fatigue scales collected at regular intervals can provide complementary subjective data.

Finally, combining multimodal approaches such as fNIRS, EEG, and behavioral performance can allow researchers to model the temporal evolution of fatigue and its neural correlates in real-time. By incorporating these measures, future n-back studies can improve their internal validity, reduce the confounding effects of fatigue, and more accurately isolate the cognitive processes underlying WM performance.

### 4.2 Validity and reliability of the n-back experiment

When assessing the validity of the n-back task, we need to consider both the face and convergent validity. Face validity is based on a subjective judgment which refers to whether the task appears to measure the cognitive ability it claims to assess. Face validity for an n-back task is generally high as the task explicitly requires participants to remember and respond to stimuli presented a certain number of steps back in a sequence, which intuitively engages WM (Kane et al., 2007). However, for convergent validity, there is a lack of convergence between n-back and other WM tasks like various span measures (operation span, reading span, symmetry span, and rotation span) due to the low correlation between n-back and these tasks (Oberauer et al., 2005; Colom et al., 2008; Shamosh et al., 2008; Jaeggi et al., 2010a; Redick and Lindsey, 2013; Jacola et al., 2014). For example, the complex span and n-back correlation *r*^+^ equals to 0.2 (Redick and Lindsey, 2013).

Due to the insufficient reliability, the n-back task is not a useful measure of individual differences in WM especially for clinical applications as it may influence the patients’ cognitive function assessments (Jaeggi et al., 2010a). The insufficient reliability of the n-back task may be derived from multiple aspects. The performance of participants may be driven by familiarity- and recognition-based discrimination processes instead of an active recall process (Smith and Jonides, 1998). In addition, participants are prone to achieving ceiling effects under low load factor conditions and floor effects by adding a mere amount of load factor. Furthermore, the reliability of n-back experiments is also affected by the experimental environment such as the clinical environment with an in-scanner environment (Jacola et al., 2014; Li et al., 2019). Although previous studies reported weak convergent validity between the n-back task and complex span measures, recent psychometric evidence suggests that the n-back remains a valid measure of WM when construct validity is evaluated via known-groups comparisons (Hepdarcan and Can, 2025). Specifically, the task reliably differentiates younger and older adults and shows good to excellent reliability in reaction time measures, supporting its suitability for detecting group-level WM differences rather than individual difference assessment.

### 4.3 Multi-cognitive process of the n-back task

In addition to the traditional WM process, the n-back task also involves other cognitive processes that will induce conflict. For example, the measure of WM with n-back may induce conflict with familiarity and recollection process when the current stimulus matches a previous stimulus, but not the one n items back in the sequence. Furthermore, the n-back paradigm involves a binding process, which means that the memory content is bound to the chronological order (Oberauer et al., 2005; Jaeggi et al., 2010a). Furthermore, the n-back task also involves sustained attention and inhibitory control. Even in simple cases (e.g., A-B-A in 2-back), the participant cannot rely on familiarity alone. Instead, they must suppress the automatic familiarity response and use temporal-order binding to determine whether the current item matches the one two positions back. Therefore, correct performance depends not only on WM updating, but also on inhibitory control and temporal-sequence memory, demonstrating that the n-back task involves multiple interacting cognitive processes. Hence, the n-back task is a complex measure involving multiple processes and thus decreases its construct validity (Kane et al., 2007; Jaeggi et al., 2010a).

Participants who complete the n-back task multiple times may develop familiarity, leading to acquisition effects. Although ERP study has shown that P300 enhancements are derived from n-back training and practice, whereas N160 enhancements only originated from n-back training (Chen et al., 2019), it is difficult to interpret results accurately, as performance improvements might reflect the acquisition effect rather than the inherent enhancement of WM.

In addition, WM training based on n-back tasks does not necessarily produce transfer effects, whether they are near or far transfer effects (Chooi and Thompson, 2012; Redick et al., 2013; Thompson et al., 2013). Although some studies have shown transfer effects, possibly due to small sample sizes (Morrison and Chein, 2011; Shipstead et al., 2012; Melby-Lervåg and Hulme, 2013; Lawlor-Savage and Goghari, 2016). Meanwhile, as the n-back experiment is a multi-cognitive process, it is difficult to conduct targeted cognitive training. This multi-process nature of the n-back task reduces its construct validity, because improvements in performance may arise from strategy shifts (e.g., chunking, familiarity-based responding) rather than genuine enhancement of WM updating. This also explains why n-back training sometimes results in near-transfer effects to tasks involving similar conflict or updating demands, but fails to generalize to broader cognitive domains. Furthermore, there is no unified standard for the duration, intensity, interval, etc. of n-back training. Some studies may have only conducted short-term training, while others may have undergone several weeks of intensive training. This difference may result in inconsistent effects of Gf enhancement. Concurrently, this leads to a lack of normative data for n-back experiments (Pelegrina et al., 2015). Thus, this may limit the application scenarios of n-back experiments, especially in education, rehabilitation, and clinical settings.

## 5 Future work

At present, n-back experiments are mainly applied in laboratory environments, and although there are some gamified scenes, they are far from daily life scenarios (Jaeggi et al., 2011; Monk et al., 2011; Bernecker and Ninaus, 2021; Murtha et al., 2022). Therefore, by utilizing emerging technologies such as virtual reality (VR), more complex and realistic n-back tasks can be designed better to simulate WM usage scenarios in real life. We mentioned earlier the application of n-back experiments and social science, such as the impact of social cognition, language, social stress, etc., on WM. Cultural background plays a significant role in shaping individual perspectives, behaviors, and cognitive processes. Therefore, it is necessary to conduct n-back experiments on people from different cultural backgrounds, especially children, which may have some chain reactions, such as how culture affects personality and cognition (Sugi et al., 2020).

In addition, the n-back experimental design is also worth studying as it has created too many derivative paradigms. Therefore, we need to standardize the training duration and intensity for more reliable comparisons. At the same time, it is necessary to design effective control groups in the experiment to improve the rigor of the experimental design. Furthermore, research based on n-back experiments may require long-term tracking. The study of Gf through n-back experiments is an example that long-term tracking can evaluate the persistence of Gf and transfer effects. Finally, due to the complexity of the n-back experiment and the multi-cognitive process, there are not many practical applications of n-back, and potential application areas include education, vocational training, rehabilitation, and sports psychology, which need further development.

## 6 Conclusion

The n-back experiment remains a cornerstone in cognitive neuroscience for assessing and training WM across various domains. Its adaptability and integration with neuroimaging techniques have advanced our understanding of WM processes and their neural underpinnings. While the task has demonstrated utility in clinical and educational contexts, limitations such as inconsistent validity and reliability, as well as mixed evidence for transfer effects, warrant further exploration. Future work should focus on developing standardized protocols, leveraging emerging technologies like VR for more ecological applications, and investigating cross-cultural and long-term impacts. Addressing these challenges will enhance the utility of n-back experiments in both research and practical applications, bridging the gap between laboratory findings and real-world cognitive demands.
