# Supplementary material for: Exploring the n-back task: insights, applications, and future directions
Source: Front Hum Neurosci. 2025 Dec 5;19:1721330. doi: 10.3389/fnhum.2025.1721330 (PMC12715002; doi:10.3389/fnhum.2025.1721330)
Supplement: Supplementary file 1 [file Data_Sheet_1.docx]

Supplementary Material

# Supplementary Figures and Tables

We have added an exploratory IBMA based on seven publicly available, group-level unthresholded task-fMRI maps (2016-1-21, 2017-6-26, 2017-8-26, 2017-10-17, 2017-11-23, 2017-12-13, 2019-7-16) from NeuroVault that probe WM load, including explicit 2-back>0-back contrasts as well as closely related high-low WM contrasts. Each image was aligned to MNI space; T-maps were converted to Z; study-level Z-images were combined voxel-wise using Stouffer’s method. Conceptually, increasing n elevates WM load. Relative to 0-back, 1-back already engages bilateral parietal and lateral prefrontal regions; 2-back produces stronger and more extensive recruitment of dlPFC, IPL and dorsal ACC/pre-SMA, together with stronger default-mode deactivation. Accordingly, 2-back>1-back highlights additional load-dependent control/monitoring demands (dlPFC/dorsal midline) beyond simple maintenance (1-back>0-back).

## Supplementary Figures

**Supplementary Figure 1.** Image-based meta-analytic Z map of the working memory (WM) task (Stouffer Z; threshold Z > 3 for visualization purposes). Warm colors represent consistent activation across 7 studies/contrasts, while cool colors indicate consistent deactivation. All images are registered to MNI space, and T maps have been converted to Z maps. Notable robust convergence is observed in the bilateral dlPFC and IPL, as well as supporting activation in the midline control regions and visual/cerebellar areas., in line with coordinate-based meta-analyses of n-back/WM.

**Supplementary Figure 2.** Convergence ratio map (0–1). The value for each voxel represents the proportion of times it exceeded Z > 3 across the 7 original maps. Higher values indicate stronger consistency across studies, further quantifying the convergence shown in **Supplementary Figure 1**.

(2016-1-21). "tfMRI WM 2BK TOOL zstat1." from <https://identifiers.org/neurovault.image:3194>. (Van Essen et al., 2013)

(2017-6-26). "t-value contrast 2-back minus 0-back." from <https://identifiers.org/neurovault.image:50291>. (Egli et al., 2018)

(2017-8-26). "Working memory load of 2 faces versus 1 face - NT2_Tstat." from <https://identifiers.org/neurovault.image:53143>. (Stout et al., 2017)

(2017-10-17). "Searchlight multivariate Decoding 2: visual working memory." from <https://identifiers.org/neurovault.image:55616>. (Wu et al., 2018)

(2017-11-23). "WM task over CRT task map." from <https://identifiers.org/neurovault.image:57499>.

(2017-12-13). "WM working memory zstat1." from <https://identifiers.org/neurovault.image:58192>.

(2019-7-16). "Stimulus-specific WM information in group condition." from <https://identifiers.org/neurovault.image:132799>. (Velenosi et al., 2020)

Egli, T., Coynel, D., Spalek, K., Fastenrath, M., Freytag, V., Heck, A., et al. (2018). Identification of Two Distinct Working Memory-Related Brain Networks in Healthy Young Adults. *eNeuro* 5(1)**,** ENEURO.0222-0217.2018. doi: 10.1523/ENEURO.0222-17.2018 %J eneuro.

Stout, D.M., Shackman, A.J., Pedersen, W.S., Miskovich, T.A., and Larson, C.L. (2017). Neural circuitry governing anxious individuals’ mis-allocation of working memory to threat. *Scientific Reports* 7(1)**,** 8742. doi: 10.1038/s41598-017-08443-7.

Van Essen, D.C., Smith, S.M., Barch, D.M., Behrens, T.E.J., Yacoub, E., and Ugurbil, K. (2013). The WU-Minn Human Connectome Project: An overview. *NeuroImage* 80**,** 62-79. doi: <https://doi.org/10.1016/j.neuroimage.2013.05.041>.

Velenosi, L.A., Wu, Y.-H., Schmidt, T.T., and Blankenburg, F. (2020). Intraparietal sulcus maintains working memory representations of somatosensory categories in an adaptive, context-dependent manner. *NeuroImage* 221**,** 117146. doi: <https://doi.org/10.1016/j.neuroimage.2020.117146>.

Wu, Y.-h., Uluç, I., Schmidt, T.T., Tertel, K., Kirilina, E., and Blankenburg, F. (2018). Overlapping frontoparietal networks for tactile and visual parametric working memory representations. *NeuroImage* 166**,** 325-334. doi: <https://doi.org/10.1016/j.neuroimage.2017.10.059>.
