# Supplementary figures and images for: Exploring the n-back task: insights, applications, and future directions
Source: Front Hum Neurosci. 2025 Dec 5;19:1721330. doi: 10.3389/fnhum.2025.1721330 (PMC12715002; doi:10.3389/fnhum.2025.1721330)

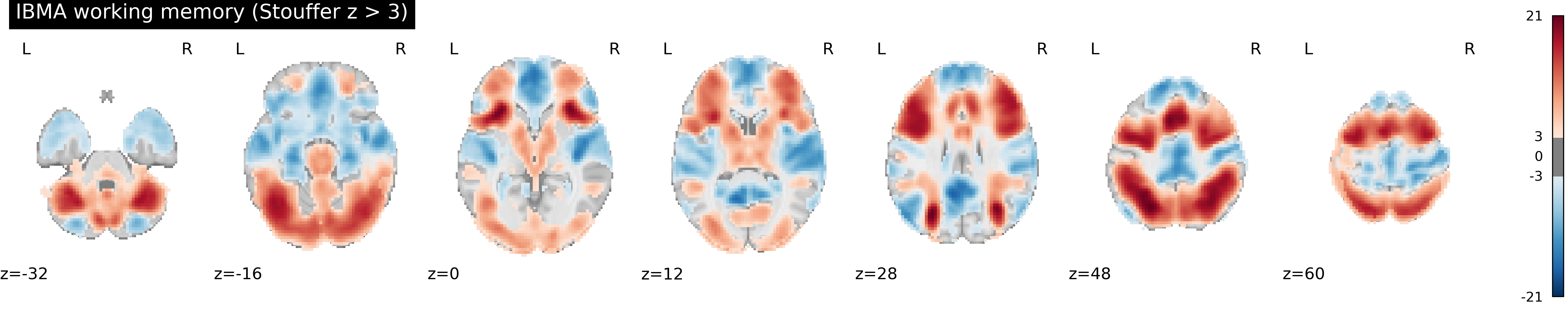

Supplement: Supplementary file 2 [file Image_1.png]

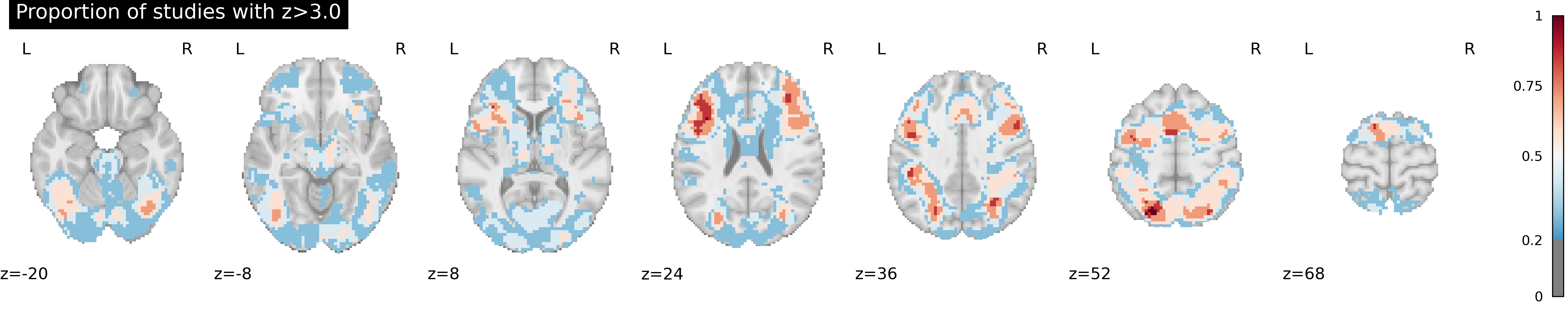

Supplement: Supplementary file 3 [file Image_2.png]
